# Cardiac amyloidosis‐A review of current literature for the practicing physician

**DOI:** 10.1002/clc.23572

**Published:** 2021-02-17

**Authors:** Samantha Ash, Eran Shorer, Devyani Ramgobin, Maique Vo, Jonathan Gibbons, Reshma Golamari, Rahul Jain, Rohit Jain

**Affiliations:** ^1^ Dept of Medicine University of Cape Town Cape Town South Africa; ^2^ Dept of Medicine Touro College of Osteopathic Medicine Middletown New York USA; ^3^ Dept of Medicine Penn State Milton S Hershey Medical Center Hershey Pennsylvania USA; ^4^ Dept of Medicine Indiana University School of Medicine Indianapolis Indiana USA

**Keywords:** AL, amyloid deposits, amyloidosis, ATTR, cardiac amyloidosis

## Abstract

The amyloidoses are a family of diseases in which misfolded precursor proteins aggregate to form amyloid and deposit in body tissues. A very serious yet underrecognized form of this disease is cardiac amyloidosis, in which amyloid deposits into the extracellular space of the myocardium, resulting in thickening and stiffening of ventricular walls with resultant heart failure and conductive dysfunction. This review provides a discussion of the pathogenesis and clinical presentation of cardiac amyloidosis subtypes, as well as an up‐to‐date approach to diagnosis and treatment. Significant progress has been made in recent years regarding diagnosis and treatment of this condition, but prognosis remains heavily reliant on early detection of the disease. Two types of precursor protein are responsible for most cardiac amyloidosis cases: transthyretin amyloid, and immunoglobulin‐derived light chain amyloid. An early diagnosis of cardiac amyloidosis can allow for novel treatment modalities to be initiated with the potential to improve prognosis.

**Abbreviations**
ACE: angiotensin converting enzyme
AL: immunoglobulin‐derived light chain amyloidosis
ATTR: transthyretin amyloidosis
BNP: B‐type natriuretic peptide
cTnT: cardiac Troponin‐T
FLC: free light chain
hATTR: hereditary ATTR
HfpEF: heart failure with preserved ejection fraction
IFE: immunofixation electrophoresis
NT‐proBNP: N‐terminal proBNP
SAA: serum amyloid A
TTR: transthyretin
wtATTR: wild type ATTR

## 1 INTRODUCTION

“Amyloidosis” refers to a group of diseases which are frequently underrecognized and misdiagnosed, resulting in inadequate management and inefficient resource utilization. These diseases differ in etiology, (and to a degree, presentation) but share a common gross and microscopic appearance.^1^

Amyloidosis is the consequence of the extracellular deposition of amyloid fibrils (aggregates of insoluble low molecular‐weight protein subunits). The deposition of amyloid can occur at a variety of sites, the particulars of which are determined by the etiology of the amyloidosis.^2^

Several types of amyloidosis have been described based on the specific protein comprising the deposited fibrils (Table 1). The most pertinent forms of the disease in the setting of cardiac amyloidosis are transthyretin amyloidosis (ATTR) and immunoglobulin‐derived light chain amyloidosis (AL). It is uncommon for secondary (AA) amyloidosis to affect the heart and this is rarely seen in developed countries where severe chronic inflammatory processes are generally well managed.^3^

**TABLE 1:** Type of amyloidosis characterized by precursor protein, cardiac and extracardiac manifestations

Amyloid type	AL	hATTR	ATTRwt	AA (secondary)
Precursor protein	Monoclonal light chains	Mutated transthyretin	Normal Transthyretin	Serum amyloid A
Sex	Males (>60%)^10^	Males (76–86%)^14^	Males (90%)^7^	Either
Typical Age	>50 years^3^	>50 years^10^	>65 years^3^	> 20 years^3^
Cardiac manifestations	Right‐sided HFpEF.(more severe than ATTR)^8^ Usually sinus rhythm^4^ Can have atrial/ventricular arrhythmias. First/second degree or advanced heart block.^10^ Severe hypotension with ACE inhibitor use.^3^ Vascular involvement not uncommon.^6^	Right‐sided HFpEF. Atrial/ventricular arrhythmias. First/second degree or advanced heart block.^10^ Pacemaker often required in Val30Met mutation.^5^	Right‐sided HFpEF. More conduction issues that hATTR (A. Fib).^9^ First/second degree or advanced heart block.^10^	Uncommon but can have ventricular wall thickening with right‐sided HFpEF.^11^
Extracardiac manifestations	Multiorgan involvement. Nephrotic syndrome (most common)^3^ Hepatomegaly/Splenomegaly^11^ Periorbital bruising (“panda eyes”)^4^ Macroglossia Nail dystrophy Submandibular gland enlargement.^1^ Peripheral polyneuropathy and autonomic neuropathy^10^ Cerebral involvement does not occur^3^ Carpal Tunnel Syndrome^4^	Depends on specific mutation. May be cardiac‐predominant, neuropathy‐predominant, or mixed.^3^ May have a sensorimotor polyneuropathy, or an autonomic neuropathy.^7^ May include kidney (uncommon), ophthalmological (vitreous deposition),^13^ and musculoskeletal involvement (Carpal Tunnel Syndrome, tendon rupture, lumbar spinal stenosis)^14^	Typically an isolated cardiomyopathy.^3^ Carpal Tunnel Syndrome^3^ Lumbar spinal stenosis^39^	Underlying chronic inflammatory process.^3^

Abbreviations: ACE, angiotensin converting enzyme; AL, immunoglobulin‐derived light chain amyloidosis; ATTR, transthyretin amyloidosis; hATTR, hereditary ATTR; wtATTR, wildtype ATTR; HFpEF, heart failure with preserved ejection fraction.

Cardiac amyloidosis most commonly occurs in the setting of systemic amyloidosis with multi‐organ involvement, although isolated cardiac amyloidosis has been described. Cardiac amyloidosis can progress quickly with rapid myocardial wall thickening and progression to congestive cardiac failure.^4^

Despite the ongoing development of new treatment modalities, the prognosis of amyloidosis (especially with cardiac involvement) remains poor. This is particularly the case when the diagnosis is missed on first presentation and is only determined once the myocardium has suffered irreparable damage, as the patient is often too unwell to survive treatment.^4^ Therefore an understanding of cardiac amyloidosis and a high index of suspicion is essential in the improvement of the prognosis of these patients.

## 2 PATHOGENESIS OF CARDIAC AMYLOIDOSIS

The clinical syndrome of cardiac amyloidosis is the consequence of extracellular deposition of proteins which have folded and aggregated such that they form amyloid fibrils.^5^

Proteins fold inappropriately for a multitude of reasons. Some proteins have an inherent propensity to misfold and deposit when in high concentrations or when the patient ages (as is the case with transthyretin), while others misfold due to an alteration in the encoding genes of the protein. Alternatively, abnormal proteolytic remodeling causing a conformational change to the precursor protein may make it more likely to fold inappropriately.^2^, ^6^ The amyloid fibrils formed by misfolded proteins are non‐branching, and this distinctive trait is important in discerning amyloid from other extracellular fibrils of similar size (such as collagen).^6^ The fibrils display a cross‐beta‐sheet super secondary structure which allows for their staining with Congo Red Stain.^2^

### 2.1 Pathogenesis of ATTR

**FIGURE 1:**
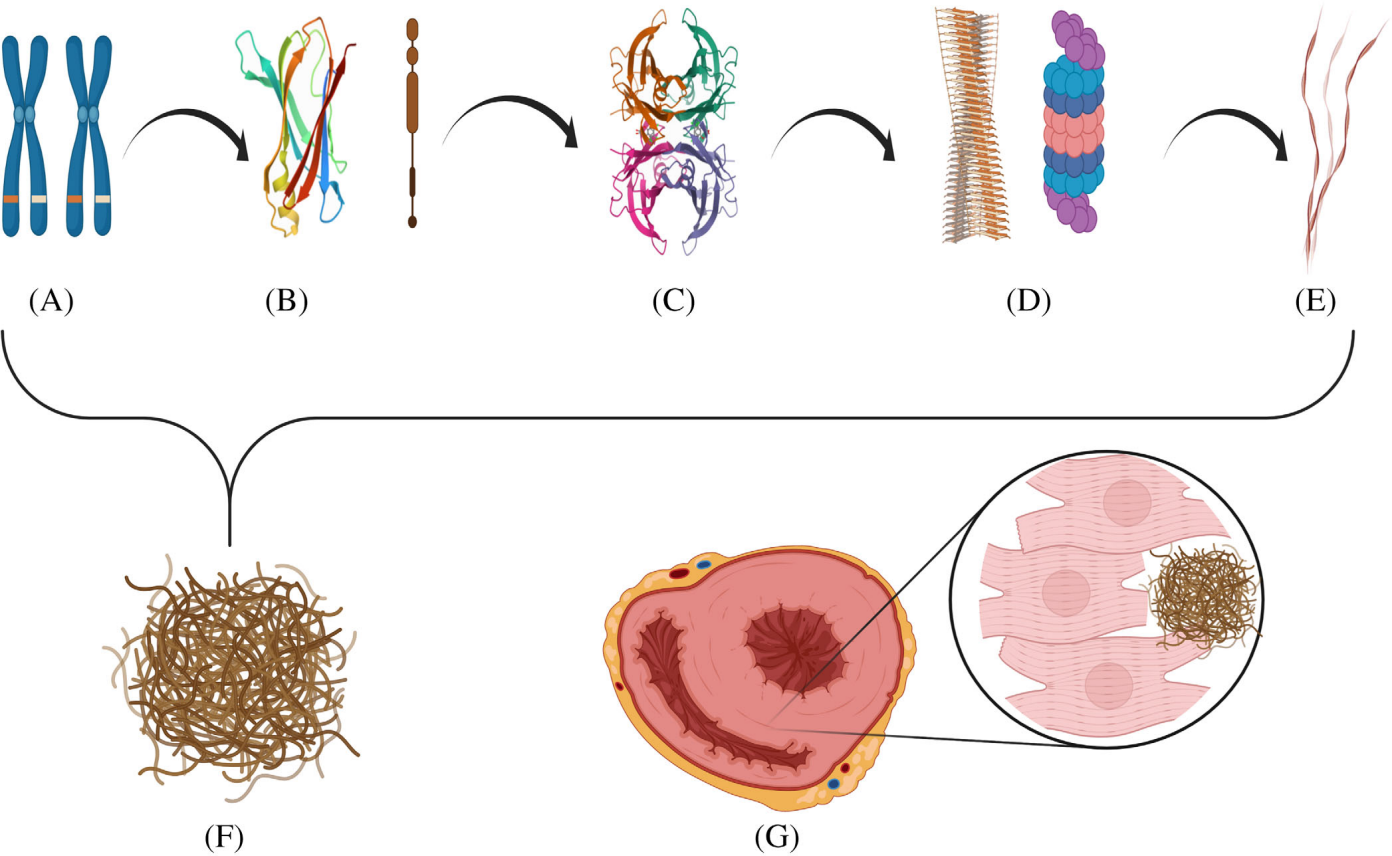
Amyloid fibril development, aggregation, and accumulation is extracellular space of myocardium with resultant cardiomegaly. Amyloid formation can result from errors in steps a‐e: a Genes encoding for Transthyretin Protein on chromosome 18; b Synthesis of Transthyretin monomer; c Transthyretin tetramer formation; d Dissociation of Transthyretin tetramers (and monomers) with subsequent proteolytic cleavage leading to the formation of Amyloid fibril; e Amyloid beta fibers composed of Amyloid fibrils; f Amyloid fibers aggregate to form Amyloid; g Cardiomegaly as a result of amyloid deposition in the extracellular space of the myocardium

ATTR is a condition in which transthyretin, a physiological protein primarily synthesized by the liver, misfolds into insoluble B‐pleated sheets and deposits as amyloid in the extracellular space of the myocardium.^7^ Transthyretin (TTR) is always present in serum and its physiological role is the transportation of retinol and thyroxine. The inherent propensity of TTR to fold and aggregate to form insoluble amyloid fibers can be increased by a single point mutation, as is the case in hereditary ATTR (hATTR)^7^ (Figure 1). “Wild type” ATTR (wtATTR) is similar to hATTR, except that it is non‐hereditary (sporadic) and the precursor protein is structurally normal TTR. It was formerly known as “senile systemic amyloidosis” and almost exclusively affects men over the age of 60 years.^8^

hATTR is a rare autosomal dominant condition in which a mutation on the transthyretin gene causes increased propensity of transthyretin monomers to misfold and aggregate as amyloid.^9^ The different mutations of this gene vary in penetrance and to a degree, clinical presentation.^4^ The most common variations associated with cardiac involvement are Val122ile (V122I or pV142I), Val30Met (V30M or pV50M), and Thr60Ala (T60A or pT80A).^5^ While Val30Met is the most common mutation in the rest of the world, the USA sees far more cases of Val122Ile (although it is difficult to know whether this is accurate or the result of underdiagnosis of the Val122ile mutation in the rest of the world).^9^

### 2.2 Pathogenesis of AL

Light chain amyloidosis is almost exclusively seen in individuals over the age of 40 years and does not show any sex predilection.^6^ It is the consequence of a plasma cell dyscrasia which may occur in isolation (as in primary AL) or be associated with multiple myeloma, B‐cell lymphoma, Waldenström macroglobulinemia, and other plasma cell dyscrasias.^10^, ^11^ In primary AL, a monoclonal dominance of a light chain isotype is evident in 5–10% of bone marrow plasma cells.^11^ This monoclonal plasma cell dyscrasia results in the overproduction of abnormal lambda or kappa light chains, which become insoluble following misfolding and deposit in the tissues.^3^ In primary AL, lambda free light chains predominate over kappa free light chains (3:1), whereas in multiple myeloma and other plasma cell dyscrasias, kappa free light chains tend to predominate, in a lambda‐to‐kappa ratio of 1:2.^11^

Infiltration of cardiac structures is postulated to damage the tissues in two ways: first, AL deposits in the extracellular space of the myocardium and coronary blood vessels which results in cardiomyocyte necrosis and interstitial fibrosis (as is the case in other varieties of amyloidosis).^8^ Second, it is thought that oxidative stress due to circulating light chain toxicity is directly myotoxic—which is unique to AL.^6^

### 2.3 Pathogenesis of AA amyloidosis

The precursor protein for secondary amyloidosis is serum amyloid A, an inflammatory protein. Unsurprisingly, secondary amyloidosis most commonly occurs in the context of a chronic inflammatory process such as rheumatoid arthritis, or in a chronic infective setting such as tuberculosis, bronchiectasis, or leprosy (although an underlying inflammatory disorder is not mandatory). For this reason, secondary amyloidosis is uncommon in developed countries. Furthermore, secondary amyloidosis usually affects the kidneys, and myocardial involvement is rare.^4^

## 3 CARDIOVASCULAR MANIFESTATIONS OF AMYLOIDOSIS

**FIGURE 2:**
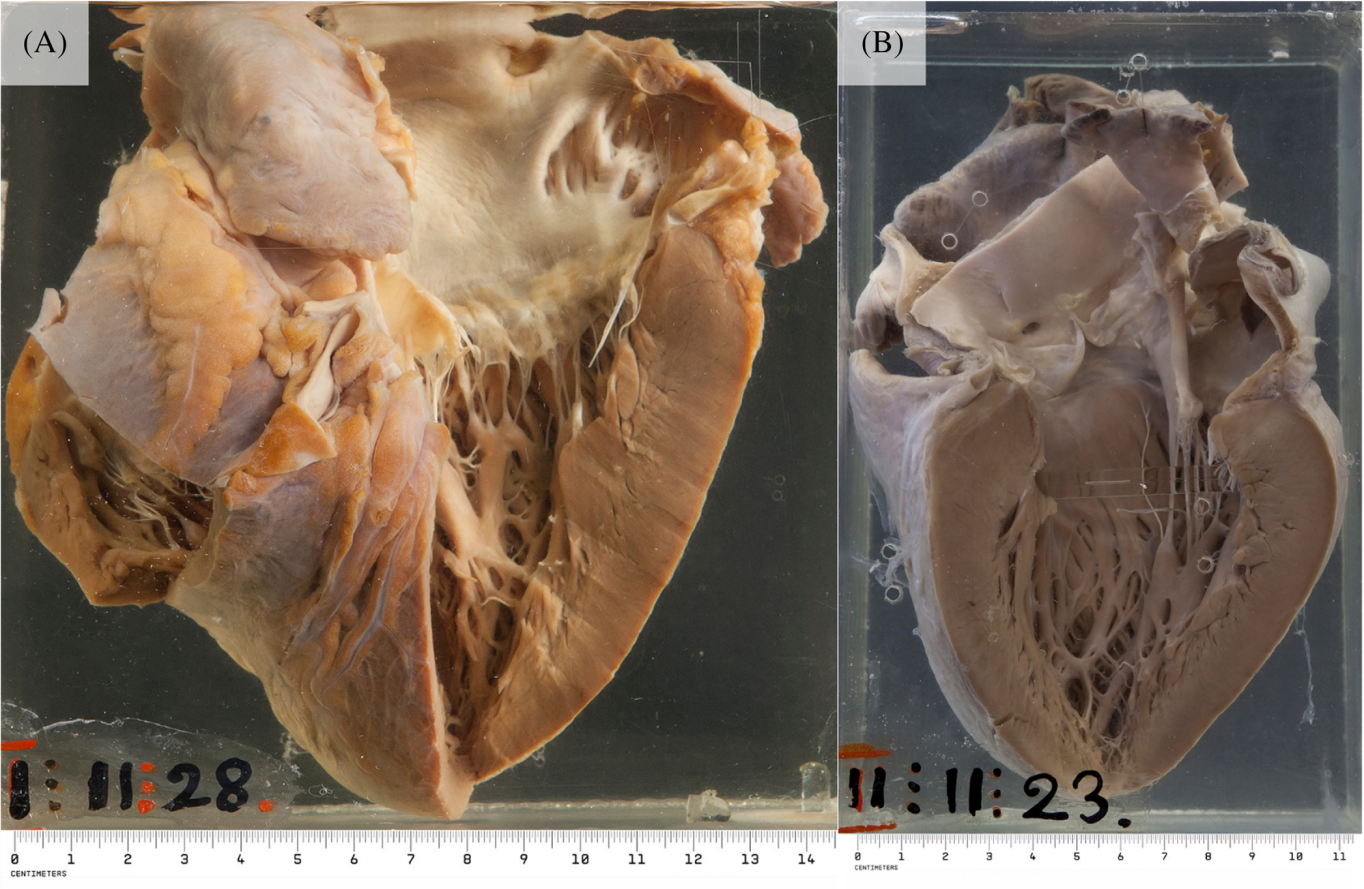
(A) An enlarged heart with a firm and slightly translucent myocardium (black arrows) and a granular appearance to the left atrial endocardium (white arrow) indicative of amyloid deposition. (B) A specimen showing significant thickening of the myocardium of all four chambers, with the typical waxy translucency of amyloid (black arrows). Macroscopic images courtesy of Dr Jane Yeats, Mr Jurgen Geitner, Pathology Learning Centre, University of Cape Town

Amyloid can deposit into any cardiac structures, including the endocardium, valves, myocardium, epicardium, and parietal pericardium.^6^ The main pathology in cardiac amyloidosis is biventricular thickening and stiffening causing a restrictive cardiomyopathy with diastolic dysfunction (HFpEF) (Figure 2).^10^ This is the consequence of amyloid infiltration into the myocardium which causes changes in calcium transport, receptor modulation, cellular metabolism, and cardiomyocyte oedema.^11^

Cardiac amyloidosis presents as heart failure with preserved ejection fraction (HFpEF), however, reduced left ventricular end diastolic volume and impaired diastolic functioning results in diminished stroke volume and cardiac output.^12^

Although amyloid deposits throughout both ventricles, signs of right heart failure predominate, with findings of pedal edema, raised jugular‐venous pressure, ascites, and hepatomegaly being evident.^7^ The apex is generally not displaced and may be impalpable in advanced disease. A third heart sound (S3) may be audible in advanced right heart failure. Despite the presence of a restrictive cardiomyopathy, a fourth heart sound is almost never heard due to diminished atrial kick as a result of amyloid infiltration in the atria.^10^

Deposition of amyloid into the atria is common and tends not to be extensive. Furthermore, atrial dilatation develops as a consequence of raised left ventricular filling pressures. This occurs due to interstitial amyloid deposition which causes a restrictive cardiomyopathy.^6^

The most common arrhythmia found in cardiac amyloidosis is atrial fibrillation, although complex ventricular arrhythmias are also seen. First degree, second degree, or advanced heart block have also been described, as well as sudden cardiac death.^10^ Patients may also present with angina or myocardial infarction due to deposition of amyloid in the coronary arteries.^1^

### 3.1 Cardiovascular manifestations of AL

AL almost invariably involves the cardiovascular system, with approximately 90% of cases involving the heart. While infiltration of any cardiac structure with amyloid is possible in AL, vascular deposition is more commonly seen in this form of the disease than in ATTR.^6^ Importantly, heart failure due to AL is more severe than that ATTR despite ATTR causing more significant left ventricular hypertrophy (this speaks to the postulated role of oxidative stress due to specific circulating light chains in AL.^8^

In addition to diastolic dysfunction, AL can also manifest as a rhythm disturbance due to amyloid deposits in the conduction system (with sinoatrial fibrosis or atrioventricular fibrosis).^11^ While atrial fibrillation can develop as the disease progresses, the rhythm is usually sinus—it is postulated that this could be due to extensive involvement of the atria resulting in loss of functioning atrial myocardium to sustain fibrillatory wavelets for atrial fibrillation to occur.^4^

### 3.2 Cardiovascular manifestations of ATTR

ATTR and AL both tend to present as HFpEF with signs of right heart failure;^13^ however, cardiac involvement is rarer in ATTR than in the latter.^11^ The cardiovascular presentation of hATTR varies according to the causative genetic mutation.^11^ For example, those with the Val30Met transthyretin mutation commonly have conduction issues requiring pacemaker placement, while other variants such as Val122Ile and Thr60Ala (T60A) commonly affect the cardiovascular system, but do not primarily affect the conduction system.^5^

In comparison to wtATTR, those with the Val122Ile mutation have a worse New York Heart Association functional class with a lower quality of life (indexed by EQ‐5D), but there is no difference in overall survival between these two forms of ATTR.^9^ Importantly, those with wtATTR are more likely to have rhythm disturbances (typically atrial fibrillation) than those with hATTR.^9^

A useful flag for suspecting ATTR is hypertension that resolves over time, and an intolerance of angiotensin receptor blockers, angiotensin converting enzyme inhibitors, or beta blockers.^13^

### 3.3 Cardiovascular manifestations of secondary amyloidosis

Patients with secondary amyloidosis are less likely to have cardiac involvement than those with other forms of the disease When present, cardiac manifestations include severe ventricular wall thickening with resultant motion abnormalities.^11^

## 4 EXTRACARDIAC MANIFESTATIONS

Systemic manifestations amyloidosis, which may precede cardiac involvement subsequent heart failure provide an invaluable window of opportunity for early diagnosis and intervention.

### 4.1 Immunoglobulin‐derived light chain amyloidosis

AL is frequently a disease of multi‐organ involvement, with nephrotic syndrome being most common, followed by cardiac involvement.^3^ Occasionally, patients present with hepatomegaly or splenomegaly,^11^ and weight loss and fatigue are common.^1^ Infiltration of the soft tissues and small vessels may be evidenced by macroglossia, periorbital purpura (so‐called “panda eyes”), nail dystrophy, and submandibular gland enlargement.^1^, ^4^ Involvement of the peripheral nervous system is quite common, typically manifesting as a sensorimotor neuropathy in a glove‐and‐stocking distribution.^1^ The central nervous system, however, is not affected.^3^ Carpal Tunnel Syndrome is also associated with AL.^4^ Autonomic neuropathy is a useful diagnostic clue, and causes orthostatic hypotension, fluctuating changes to bowel habits, and erectile dysfunction.^1^

### 4.2 Hereditary ATTR

The phenotype hATTR tends to be either cardiac‐predominant or neuropathy‐predominant.^3^ This is determined by the site of an amino acid substitution on the TTR gene.^1^ The typical pattern of hATTR amyloid neuropathy is an ascending symmetrical length‐dependent sensorimotor axonal polyneuropathy.^7^ This can have a major effect on quality of life. Interestingly, those with the Val122ile mutation have more severe neurological symptoms and walking disability than those with wtATTR.^9^ As in AL amyloidosis, hATTR is also associated with autonomic neuropathy, which primarily presents with gastrointestinal symptoms.^7^ Furthermore, Carpal Tunnel Syndrome, tendon rupture, and lumbar spinal stenosis are all associated with hATTR.^13^ Occasionally, patients have ophthalmological involvement in the form of vitreous deposition.^14^ Unlike in AL amyloidosis, macroglossia does not occur in hATTR, and renal involvement is less common.^11^

### 4.3 Wildtype ATTR

In wtATTR, the heart is usually the only clinically affected organ, but signs of heart failure may be preceded by lumbar spinal stenosis or bilateral Carpal Tunnel Syndrome by 10–15 years.^1^

## 5 SECONDARY AMYLOIDOSIS

Secondary amyloidosis is rare, and when it occurs, signs of the instigating chronic inflammatory process may be apparent.^4^ Examples of possible causative inflammatory diseases are rheumatoid arthritis, inflammatory bowel disease, familial Mediterranean fever, chronic lung diseases, tuberculosis, and leprosy.^11^

## 6 DIAGNOSIS

The gold standard for diagnosis of cardiac amyloidosis is the demonstration of apple‐green birefringence in polarized light microscopy of Congo Red stained tissue; however, less invasive techniques can be used to raise the index of suspicion of this diagnosis.

### 6.1 Serum biomarkers

#### 6.1.1 Non‐specific serum biomarkers

B‐type natriuretic peptide (BNP) and N‐terminal proBNP (NT‐proBNP) are raised in all cases of heart failure, but may be disproportionately high in cardiac amyloidosis due to direct compression of cardiomyocytes and stress caused by raised filling pressures.^3^ Thus these can be useful in detecting cardiac involvement in systemic amyloidosis or in evaluating the severity of disease. Additionally, serial NT‐proBNP measurements are useful in the evaluation of post‐chemotherapy prognostic outcomes.^10^ Cardiac Troponin‐T (cTnT), another reliable indicator of cardiomyocyte death, is a useful negative prognostic indicator in both AL and ATTR cardiac amyloidosis.^15^

#### 6.1.2 AL‐specific biomarkers

The detection of a monoclonal gammopathy is achieved with the use of serum and urinary quantitative free light chain (FLC) measurements and immunofixation electrophoresis (IFE).^15^ The ratio of kappa/lambda free light chains must be calculated. An abnormal kappa lambda ratio is <0.26 or > 1.65, and is present in more than 90% of untreated AL cases.^3^, ^5^Importantly, raised FLC and IFE are not specific markers of AL, as they are sometimes raised in wtATTR and cases of monogammopathy of undetermined significance. Therefore, where there is concern of this ambiguity, more specific testing is needed to determine the composition of the amyloid.^15^ A bone marrow biopsy is recommended in all cases of suspected AL to assess the percentage of plasma cells in order to rule out multiple myeloma and other rare hematological causes of amyloid deposition.^3^ Mayo Clinic has developed a staging system for AL which incorporates NT‐proBNP, Troponins, and dFLC which can be used for prognostic evaluation.^16^

### 6.2 Electrocardiography

The hallmark ECG finding of cardiac amyloidosis is low voltage QRS complexes in the limb leads, with poor R‐wave progression in the precordial leads.^11^ Whilst helpful when present, a lack of this finding should not alleviate suspicion of cardiac amyloidosis, as only 40% of cases with biopsy‐proved ATTR have low‐voltage ECGs.^5^ However, the prevalence of low‐voltage ECGs depends greatly on the definition used ‐ as such, a cut‐off of </=24.5 mm QRS amplitude in limb leads provides 80% specificity and 58.72% sensitivity in detecting AL with cardiac involvement.^17^ Thus the utility of a low voltage ECG as a screening tool is limited by its low sensitivity.^13^

Despite greater cardiac infiltration of amyloid in ATTR, the finding of low ECG voltage is far more common in AL.^3^ Furthermore, patients with the Val122ile mutation are more likely to have a low voltage ECG than those with wtATTR, but this finding remains uncommon in both forms of ATTR.^9^

In fact, a pseudoinfarct pattern is a commoner ECG finding in cardiac amyloidosis than low QRS voltages, and is more common in AL amyloidosis than in ATTR.^4^, ^9^ ECG signs of infarction with or without coronary obstruction are likely a result of amyloid deposition in the smaller intramyocardial arteries and microcirculation.^11^

Unlike QRS complexes in cardiac amyloidosis, P waves have normal voltage but are prolonged with morphological abnormalities, indicative of inter or intra‐atrial conduction delay secondary to amyloid deposition.^3^ Additionally, atrial fibrillation is a common finding, particularly in AL and wtATTR.^11^ Other conduction abnormalities occur, including varying degrees of atrioventricular blocks and bundle branch blocks.^18^

### 6.3 Echocardiography

Cardiac amyloidosis causes concentric bi‐ventricular wall thickening which is often in excess of 15 mm, with wall thickness greater than 18 mm being far more common in ATTR than in AL.^3^ (Figure 2). A finding of increased ventricular wall mass in the setting of a low voltage ECG should raise suspicion of cardiac amyloidosis, as the thickening is due to amyloid infiltration and not true cardiomyocyte hypertrophy.^14^ Despite wtATTR causing statistically significant greater ventricular wall thickening on a population level, this cannot be used to differentiate it from hATTR or AL on an individual level.^19^

Importantly, echogenicity in cardiac amyloidosis is increased far more that what would be expected in true ventricular hypertrophy, and the myocardial texture has a typical “granular sparkling” appearance.^3^, ^11^

Bi‐atrial enlargement and atrial septal thickening are commonplace.^18^ Furthermore, the atrium can become a site of thrombus formation (even in the absence of arrhythmia) as a result of low stroke volume and an irregular atrial endocardial surface due to amyloid infiltration.^3^, ^20^

Another frequent finding in cardiac amyloidosis is valvular leaflet thickening, although this is usually mild and of little consequence. Notably, if a pacemaker has been placed the thickened amyloid‐infiltrated tricuspid leaflets may not mold around the wire, and even a small amount of regurgitation can significantly increase pressures due to a stiff right atrium.^3^ Occasionally, pleural or pericardial effusions are seen.^5^

On echocardiogram, strain imaging reveals impaired longitudinal strain in basal and midventricular segments with sparing of apical segments which is characteristic of amyloidosis.^18^ This can be an invaluable early sign, as it plots in a “bulls‐eye” pattern, which is a rare finding in other cardiomyopathies.^21^

### 6.4 Cardiovascular magnetic resonance imaging

Cardiovascular magnetic resonance imaging (CMR) has great utility in cardiac amyloidosis both as a screening tool and as a novel means by which to track response to treatment.^1^

As seen on CMR, concentric left ventricular hypertrophy is the most common form of remodeling seen in AL, and asymmetric septal hypertrophy is the most common in ATTR.^21^ A very common finding is disproportionate biatrial enlargement with atrial septal wall thickening, though this is not specific to cardiac amyloidosis.^22^

Deposition of amyloid fibrils in the myocardium results in raised extracellular volume which is evidenced by late gadolinium enhancement (LGE). A diffuse subendocardial pattern of LGE is essentially pathognomonic of cardiac amyloidosis (specificity 95%),^23^ although diffuse transmural LGE is most common.^22^ Furthermore, diffuse transmural LGE is more common in ATTR than in AL, whilst a diffuse subendocardial pattern is more common in AL amyloidosis.^22^ Furthermore, the signal of hearts infiltrated by amyloid cannot be suppressed with phase sensitive recovery LGE.^18^

Native T1 is elevated in cardiac amyloidosis, as is post‐contrast extracellular volume fraction (ECV).^13^, ^18^ ECV is thought to be a more reliable means of quantification of myocardial amyloid burden, and may allow for better prognostic evaluation and treatment response tracking.^1^, ^22^

### 6.5 Nuclear imaging

Technetium Pyrophosphate Scintigraphy (PYP scan) is a nuclear imaging study which detects cardiac transthyretin and can be used (in conjunction with other clinical investigations) to diagnose TTR amyloidosis.^15^ Due to its less invasive nature compared to cardiac biopsies this method is preferred, although does involve exposure to ionizing radiation. PYP scans require the injection of a radiotracer (Technetium Pyrophosphate, referred to as TC‐PYP) into the venous circulation. Other tracers such as Tc‐DPD and Tc‐HMDP are also used but are less common.^13^

Once injected, the radiotracer binds to TTR amyloid fibrils.

PYP scans hold major advantages over other imaging modalities. First, PYP scans are able to detect TTR fibrils in the heart prior to the onset of cardiac hypertrophy and electrophysiological changes. This allows PYP scans to pick up ATTR before echocardiographic and other similar investigations which rely on identifying structural or voltage changes in the heart.^13^ Second, in the setting of normal serum kappa/lambda ratio and immunofixation electrophoresis, scintigraphy can accurately differentiate ATTR from AL cardiac amyloidosis—a feat that cannot be accomplished by MRI or echocardiography alone.^5^ Lastly, PYP scans are able to prognosticate in ATTR amyloidosis. A heart to contralateral (H/CL) ratio of 1.6 or greater is associated with significantly worse outcomes over 5 years.^24^

In a multicentre trial PYP scintigraphy had a sensitivity of 91% and specificity of 92% in identifying ATTR amyloidosis.^24^ However, the sensitivity of scintigraphy is highly dependent on whether the patient has a monoclonal gammopathy (which can be present in up to 40% of patients with ATTR). In the setting of monoclonal gammopathy of unknown significance, scintigraphy cannot be used in isolation to diagnose ATTR. In these instances, myocardial biopsy is necessary to provide a diagnosis at ATTR cardiomyopathy.^13^

### 6.6 Biopsy

Endomyocardial biopsy and histological analysis is the gold standard for identifying cardiac amyloidosis, however biopsies are invasive, require substantive technical expertise and pose a risk of complication (albeit small).^13^ To mitigate some of these concerns, abdominal fat pad and bone marrow biopsies may be performed. Fat pad fine needle aspiration is sensitive in detecting systemic AL (sensitivity 84%), but has a low sensitivity in cases of hATTR and wtATTR (sensitivity 45% and 15%, respectively).^25^ This is in stark contrast to endomyocardial biopsies with Congo red staining which have a 100% sensitivity and specificity rate.^13^ Therefore, a negative biopsy of an unaffected organ should not discount a diagnosis of amyloidosis, and another biopsy should be performed ‐ in clinically suspected cardiac amyloidosis, this should be an endomyocardial biopsy.^13^

**FIGURE 3:**
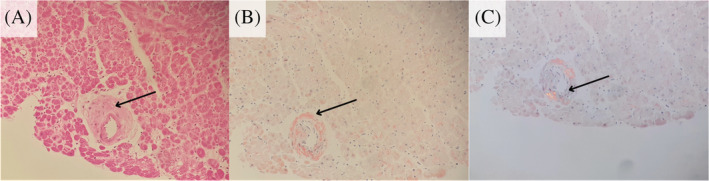
Microscopy and special staining demonstrating cardiac amyloidosis. (A) Hematoxylin and eosin stained section of a cardiac biopsy sample showing an amorphous eosinophilic deposit in a perivascular distribution. (×200 objective magnification). (B) Congo Red stained section showing a salmon‐pink color within the perivascular deposit (200x objective magnification). (C) Congo red stained section with polarized microscopy highlighting apple‐green birefringence within the perivascular deposit. (×200 objective magnification). Microscopic images courtesy of Dr Riyaadh Roberts, Division of Anatomical Pathology, University of Cape

The diagnosis of amyloidosis is confirmed on Congo Red staining of affected tissue, which demonstrates a yellow‐green birefringence under polarized light.^11^ Histologically, amyloid can deposit in a pericellular pattern, a nodular mattern, or mixed.^6^ Interestingly, the deposition pattern in AL is usually a diffuse pericellular infiltration with deposition in small blood vessels, while that of ATTR is usually nodular.^3^ (Figure 3).

Once the diagnosis is confirmed, the specific amyloid protein can be typed using mass spectrometry (preferable if available), or immunohistochemistry.^13^

### 6.7 Genetic testing

**FIGURE 4:**
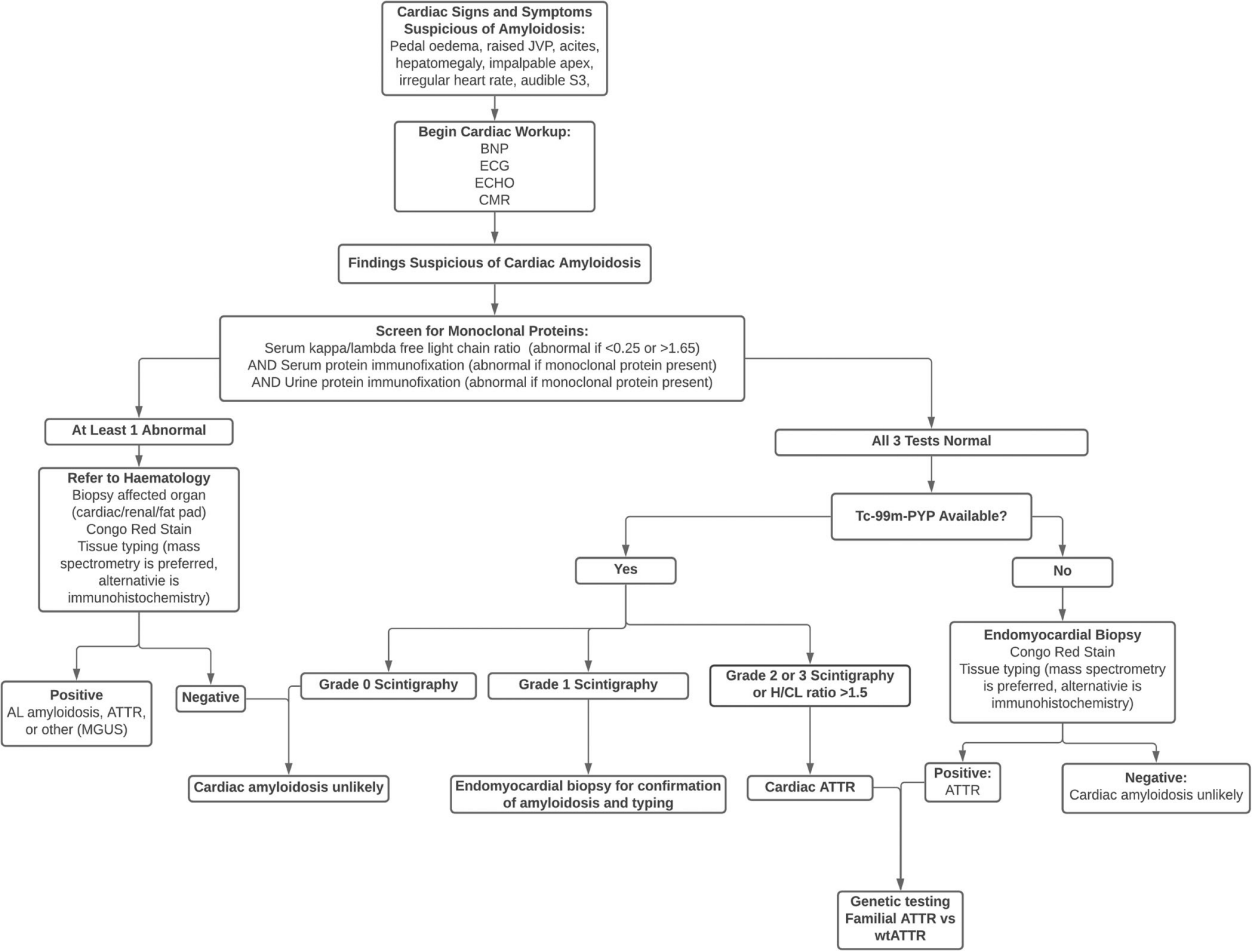
Algorithm for the investigation of suspected cardiac amyloidosis. The above algorithm is based on the work of Maurer et al, 2019; Kittleson et al, 2020; Yamamoto and Yokochi, 2019; and Bhogal et al, 2017

Once ATTR has been proven (positive scintigraphy or cardiac biopsy), genotyping is needed in order to discriminate between wild type (wtATTR) and hereditary variants (hATTR). Subtypes of hATTR demonstrate variable penetrance thus a diagnosis of hATTR cannot be excluded without genetic testing, even in the absence of relevant family history. Discriminating between wtATTR and hATTR is essential as definitive diagnosis of hATTR allows for genetic counseling and disease‐specific treatment programmes.^13^ For instance the Val122IIe mutation, an hATTR subtype, is highly aggressive and therefore necessitates unique therapeutic and follow up regimens.^5^ The staging system developed by Grogan et al. (2016)^26^ requires discrimination betweeen wtATTR and hATTR, however a newer staging system described by Gillmore et al. (2018)^27^ stages ATTR irrespective of whether the wild type or other variants are present (Figure 4).

## 7 TREATMENT

Management of cardiac amyloidosis is complex and specific for the type of amyloidosis that affects the patient. AL is preferentially treated with stem cell transplants, chemotherapy, and proteasome inhibitors. The goal of treatment for AL is to reduce the production of light chains, remove light chain amyloid deposits and inhibit amyloid fibril formation. The current standard of care for AL patients is chemotherapy using Cyclophosphamide, bortezomib and dexamethasone (CyBorD).^28^ In a phase 3, ANDROMEDA study, Daratumumab, (DARA‐SC), a drug used in treatment of multiple myeloma, was studied in conjunction with CyBorD. This demonstrated robust hematologic and organ responses.^29^ In patients with cardiac involvement of amyloidosis, the median time to response was 114 days. Among those with cardiac involvement, responses were seen in 9 of 17 patients (53%). The overall organ response rate, including cardiac, kidney and liver, was 64% at 17.3 months. The Andromeda study effectively demonstrated that CyBorD coupled with Daratumumab works effectively to produce responses in patients with AL amyloidosis. Therefore, it is likely that the current standard of care for AL treatment will change to include Daratumumab as a first line treatment. Similarly, oral treatment with proteasome inhibitor Ixazomib resulted in a 52% hematologic response rate and 56% organ response in relapse‐refractory AL patients.^30^ While many studies assess the hematologic response and organ responses, such as the kidney to treatment, there remains much to be explored regarding cardiac response.

Conversely, ATTR treatment is focused on stabilizing the transthyretin tetramers and removing transthyretin amyloid deposits. Drugs such as Tafamidis and Diflusinal focus on stabilizing the TTR tetramer in order to prevent disassociation and ATTR fibril formation.^31^, ^32^ Doxycycline and tauroursodeoxycholic acid result in separation of amyloid deposits as well as decrease the accumulation of toxic TTR aggregates.^33^ In patients with hereditary transthyretin mediated amyloidosis, another avenue taken is by silencing the gene and decrease the production of TTR by the liver. Patisiran, is a double stranded small interfering RNA (siRNA) that targets TTR messenger RNA (mRNA) to decrease liver production of TTR. The effect of Patisiran is comparable to ATTR stabilizers in that it can improve polyneuropathy as well as prevent cardiac decline in patients with hereditary ATTR.^34^ Supportive treatments are also used in conjunction to provide symptom relief from heart failure and conduction dysfunction. The treatment of patients with cardiac amyloidosis‐induced heart failure differs from the ordinary treatment of patients with heart failure. Many drugs that are commonly prescribed to treat heart failure have been proven to be unhelpful in amyloidosis‐induced heart failure. Angiotensin‐converting‐enzyme inhibitors (ACE inhibitors), angiotensin II receptor blockers (ARBs), and beta blockers all decrease mortality in most patients with heart failure.^35^ However, in patients with amyloidosis‐induced heart failure, these drugs are detrimental. ACE inhibitors and ARBs promote hypotension due to autonomic dysfunction and can only be tolerated in low doses in patients with cardiac amyloidosis. Alike, beta blockers have been shown to provoke bradyarrhythmias in these patients.^36^ Normally, calcium channel blockers (CCBs) are known to be particularly helpful in the treatment of diastolic heart failure. Yet, in patients with cardiac amyloidosis, calcium channel blockers are ineffective due to strong binding of the drug to amyloid fibrils leading to worsening heart failure, hypotension, and syncope.^12^ Strong binding to amyloid fibrils can also occur with the use of digoxin, leading to digitalis toxicity, which includes yellow‐tinted vision, cholinergic agonism, and arrhythmias.^36^ For management of fluid overload, low sodium diet and fluid restriction is recommended as well as usage of loop diuretics and aldosterone inhibitors.^1^, ^37^ Management of heart conduction dysfunction can be managed using a pacemaker and ICD; however, studies have shown that ICD does not improve survival.^38^ Organ transplantation is a definitive treatment option for patients with cardiac amyloidosis. For AL amyloidosis a heart transplant is recommended while in ATTR, both a heart and liver transplant is needed.^22^, ^38^

## 8 CONCLUSION

Cardiac amyloidosis is a disease with a poor prognosis if diagnosed late. With a high degree of clinical suspicion and apt use of available diagnostic technologies, an early diagnosis of cardiac amyloidosis can allow for novel treatment modalities to be initiated with the potential to improve prognosis.

## CONFLICT OF INTEREST

None.

## Data Availability

Data sharing not applicable to this article as no datasets were generated or analysed during the current study.
